# Targeting SMOX Preserves Optic Nerve Myelin, Axonal Integrity, and Visual Function in Multiple Sclerosis

**DOI:** 10.3390/biom15020158

**Published:** 2025-01-21

**Authors:** Harry O. Henry-Ojo, Fang Liu, S. Priya Narayanan

**Affiliations:** 1 Program in Clinical and Experimental Therapeutics, College of Pharmacy, University of Georgia, Augusta, GA 30907, USA; hhenryojo@augusta.edu (H.O.H.-O.); fliu1@augusta.edu (F.L.); 2 Research Division, Charlie Norwood VA Medical Center, Augusta, GA 30901, USA; 3 Culver Vision Discovery Institute, Augusta University, Augusta, GA 30907, USA

**Keywords:** multiple sclerosis, optic nerve, spermine oxidase, MDL72527, myelin, vision

## Abstract

Multiple sclerosis (MS) is a highly disabling chronic neurological condition affecting young adults. Inflammation, demyelination, and axonal damage are key pathological features of MS and its animal model, experimental autoimmune encephalomyelitis (EAE). Our previous work demonstrated that inhibiting spermine oxidase (SMOX) with MDL72527, a selective irreversible pharmacological inhibitor, significantly reduced clinical symptoms, retinal ganglion cell (RGC) loss, and optic nerve inflammation in EAE mice. The present study explored the broader therapeutic potential of SMOX inhibition, focusing on myelin preservation, axonal integrity, and visual function in the EAE model. Electron microscopy of optic nerve cross-sections showed significant preservation of myelin thickness and axonal integrity due to SMOX inhibition. The quantitative assessment showed that g-ratio and axon count metrics were significantly improved in MDL72527-treated EAE mice compared to their vehicle-treated counterparts. Immunofluorescence studies confirmed these findings, showing increased preservation of myelin and axonal proteins in MDL72527-treated EAE mice compared to the vehicle-treated group. Functional assessment studies (Electroretinography) demonstrated significant improvement in RGC function and axonal conduction in EAE mice treated with MDL72527. Furthermore, SMOX inhibition downregulated the expression of galectin3 (Gal3), a mediator of neuroinflammation, indicating Gal3’s role in SMOX-mediated neuroprotection. This study provides compelling evidence for the potential of SMOX inhibition as a therapeutic strategy in multiple sclerosis and other demyelinating disorders.

## 1. Introduction

Multiple sclerosis (MS) is a chronic, inflammatory demyelinating disease of the central nervous system (CNS) that affects approximately 2.8 million people worldwide [1,2]. MS is characterized by inflammation, the gradual erosion of myelin sheath, axonal damage, and neuronal loss, leading to a spectrum of neurological symptoms, including motor deficits, visual impairment, and cognitive dysfunction [3,4,5,6,7]. While the exact etiology of MS remains elusive, a complex interplay of genetic susceptibility, epigenetic influences, environmental triggers, and autoimmune mechanisms contributes to its pathogenesis [8]. The visual system, including the optic nerve and retina, is often among the first regions affected by MS [9,10], resulting in optic neuritis. Optic neuritis is associated with recurrent or chronic MS and often leads to visual deficits, inflammation, and loss of retinal ganglion cells (RGCs) [2,10,11]. Hence, clinical presentation with optic neuritis in patients is considered a future indication of MS [12], as early studies have shown that 50% develop MS 15 years after onset of optic neuritis, with 72% of those showing one or more lesions on initial brain MRI scans [13]. Therefore, optic neuritis diagnosis often necessitates additional examination, including visual evoked potential tests and MRI assessments to rule out the presence of brain lesions [14]. Optic neuritis significantly impacts the quality of life in MS patients. Current treatments include high-dose corticosteroids for immediate relief and immunosuppressants for long-term management [15,16]; however, these do not resolve the underlying pathology and progressive optic nerve damage [17,18]. Therefore, assessing visual function and the underlying mechanism of optic neuritis is essential for understanding the neurodegenerative and inflammatory processes in MS, as well as for evaluating the effectiveness of new treatments [11,19]. Experimental autoimmune encephalomyelitis (EAE) is a well-established animal model that recapitulates many of the immunopathological and clinical features of MS [20,21], which has been instrumental in elucidating disease mechanisms and developing therapeutic strategies. In principle, EAE is characterized by T cell-mediated inflammation, focal demyelination, and axonal loss in the CNS, particularly affecting the spinal cord and optic nerves. Moreover, the EAE model manifests a similar pattern of blood–brain barrier disruption, immune cell infiltration, and glial activation observed in MS lesions [22,23].

Recent research studies have highlighted the potential role of polyamine metabolism in neuroinflammatory and neurodegenerative processes. Dysregulated polyamine signaling has been reported in conditions such as Alzheimer’s disease [24,25], Parkinson’s disease [26,27,28], traumatic brain injury [29,30], and Ischemic stroke [31,32,33]. Polyamines, including putrescine, spermidine, and spermine, are ubiquitous polycationic molecules involved in various cellular functions, such as cell growth, differentiation, and stress response [34,35]. Spermine oxidase (SMOX) is a FAD-dependent enzyme in the polyamine pathway that catalyzes the back-conversion of spermine to spermidine. SMOX activity generates hydrogen peroxide and acrolein, which can contribute to cellular damage. Consequently, SMOX has been implicated in mediating oxidative stress and inflammation in various pathological conditions [36,37,38,39]. Similarly, elevated levels of acrolein are reported in MS patient samples and animal models [40,41]. Given the critical role of oxidative stress and inflammation in the pathogenesis of MS and EAE, we have formerly demonstrated that SMOX inhibition could offer neuroprotective effects in EAE pathology [42] and other ocular diseases [37,42,43]. To suppress SMOX activity, our research employed MDL72527 (N1,N4-bis(2,3-butadienyl)-1,4-butanediamine), a potent and selective inhibitor of SMOX, which has shown promise in ameliorating oxidative stress and inflammation in several disease models [37,44,45,46]. MDL72527 irreversibly inactivates SMOX by forming a covalent adduct with the FAD cofactor [47]. While the impact of SMOX on crucial cellular functioning is increasingly being recognized, the extent of its role in demyelinating conditions and its implications for visual dysfunction in MS/EAE remains largely unexplored.

The aim of the present study is to explore the therapeutic potential of targeting SMOX in reducing neurodegeneration and vision loss in MS. Employing the pharmacological inhibitor MDL72527 and the EAE model of MS, the present study investigates the impact of SMOX inhibition on optic nerve myelination, axonal integrity, and changes in retinal function.

## 2. Materials and Methods

### 2.1. Animals, Induction of EAE, and Clinical Scoring

All the animal experiments implemented in this study comply with the ARVO statement for using animals in Ophthalmic and vision research. The procedures employed are approved by the Institutional Animal Care and Use Committee of Augusta University, Augusta, GA, USA (Protocol Number: 21-05-127), and the Charlie Norwood VA Medical Center, Augusta, GA, USA (Protocol Number: 2016-0823). The mice utilized in this study are Wild-type female mice (12–13 weeks old; C57BL/6J background) purchased from Jackson Laboratories (Bar Harbor, ME, USA) and maintained in our animal facility. All possible precautions were taken to ensure animal suffering and pain were avoided and/or minimized during the experimental procedures.

Chronic EAE was induced using the EAE induction kit (Hooke Laboratories, Lawrence, MA, USA, cat #EK-2110), following the method previously established in our laboratory [48]. The mice were immunized on day 0 with subcutaneous injections via the flank region with 200 μL of myelin oligodendrocyte glycoprotein (MOG_35–55_) emulsion (200 μg/mouse) along with complete Freund’s adjuvant (CFA, killed Mycobacterium tuberculosis H37Ra (final concentration 400 μg/μL)). Additionally, each mouse received 100 ng i.p. injection of pertussis toxin (PTX) in 50 μL of PBS on day 0 (1–2 h after MOG_35–55_/CFA injection) and one-day post-immunization. The control groups received the immunization with CFA but without the antigen (MOG peptide), along with two doses of PTX, given on day 0 and day 1.

EAE disease progression in the mice was evaluated daily using a blinded assessment method as previously published [42,49]. The progression of ascending paralysis was measured and scored according to the conventional six-stage grading system (0–5): 0.0 (no motor deficits), 0.5 (tip of tail limp), 1.0 (limp tail), 1.5 (limp tail and hind leg inhibition), 2.0 (limp tail and weakness of hind legs), 2.5 (limp tail and dragging of hind legs), 3.0 (limp tail and complete paralysis of hind legs), 3.5 (limp tail and complete paralysis of hind legs, unable to right itself), 4.0 (limp tail, complete hind leg and partial front leg paralysis), 4.5 (complete hind and partial front leg paralysis, no movement), and 5.0 (severe paralysis or death) [4]. Animals with paralysis of all four limbs and/or weight loss of more than 20% were sacrificed immediately. Soft food was provided in the cage for mice showing signs of paralysis. EAE mice with a clinical score of 4 or lower showing a 20% reduction in body weight (from the baseline level, recorded on day 8 post-induction) were included in the study if they regained body weight within the next 3 days. Mice were euthanized at various time points by using an overdose of ketamine/xylazine cocktail, and eyeballs or retinas/optic nerves were harvested and prepared for analysis.

### 2.2. Treatment with MDL72527

MDL72527, an inhibitor of SMOX [44], was administered intraperitoneally (i.p.) at a dosage of 20 mg/kg three times per week, similar to our earlier study [42]. Normal saline was used as the vehicle. This treatment regimen was maintained until the animals were euthanized. MDL72527 or vehicle treatment started on day 1, post-EAE induction. The experimental plan involved four mice groups: vehicle-treated control (Veh-Con, WT mice immunized with CFA with two injections of PTX and receiving normal saline, i.p.); vehicle-treated EAE (Veh-EAE, WT mice immunized with MOG peptide in CFA with two injections of PTX and receiving normal saline, i.p.); MDL72527-treated EAE (MDL-EAE, WT mice immunized with MOG peptide in CFA with two injections of PTX, and receiving MDL72527, i.p.); and MDL72527-treated control (MDL-Con, WT mice immunized with CFA with two injections of PTX, and receiving MDL72527, i.p.).

### 2.3. Electron Microscopy and Quantification of g-Ratio and Axon Count

Optic nerve samples for EM were prepared following intracardial perfusion of mice using PBS and fixed in 4% paraformaldehyde and 2% glutaraldehyde in 0.1 M sodium cacodylate buffer for 24–48 h. The samples were then processed for electron microscopy at the Augusta University histology and electron microscopy core according to established protocols [50]. Thin sections of the optic nerves were sliced and collected on copper grids, then stained with uranyl acetate and lead citrate. ON tissue was observed in a JEM 1230 transmission electron microscope (JEOL USA, Inc., Peabody, MA, USA) at 110 kV and imaged with an Ultra Scan 4000 charge-coupled device camera (Gatan, Inc., Pleasanton, CA, USA) and First Light Digital Camera Controller (First Light Imaging Corp., Cambridge, MA, USA). EM images (2040 × 2040 pixels2 covering 1752 µm^2^) were taken at 1000×. EM images were taken randomly across the entire ON cross-section, with the sum of EM image areas covering at least 10% of the ON area.

For analysis, a minimum of five EM images were included for each optic nerve. The number of axons in each image was counted and averaged across all five images to determine the axonal count for each animal and, subsequently, for each animal group. Using the same images per optic nerve, the g-ratio (ratio of inner to outer diameter of an axon) was also measured for all the samples and mice groups. The axonal count and g-ratio measurement were performed using ImageJ version 2.14.0/1.54f for Mac 64-bit (https://imagej.net/Fiji/Downloads, accessed on 14 August 2024).

### 2.4. Immunofluorescence Staining of the Optic Nerve Sections and Quantification of Fluorescence Intensity

Immunostaining was performed on optic nerve sections, as described previously [42,49]. The optic nerves were isolated carefully, fixed in 4% PFA (overnight at 4 °C), and washed in PBS for 48 h, followed by cryoprotection in 30% sucrose. The cryostat sections (10 μm) were prepared and mounted on glass slides and stored at −80 °C until used. The sections were permeabilized in 0.5% Triton X-100 (10 min) and blocked in 10% normal goat serum for 1 h at room temperature. The sections were then incubated with primary antibodies (Table 1) overnight, followed by 1 h incubation with fluorescein-conjugated secondary antibodies (Table 1), as previously described. The sections were washed in PBS and covered with mounting medium (Vector Laboratories Cat. # H-1000, Burlingame, CA, USA). The high magnification images were taken at 63× using a confocal microscope, and low magnification images at 20× (LSM 780; Carl Zeiss, Thornwood, NY, USA). The magnification of the eyepiece lens was 10×, resulting in a total magnification of 630× and 200×, respectively.

Quantification was performed using a minimum of two sections (20 µm apart) per optic nerve utilized per animal for each antibody treatment following our previous methods [42]. Three non-overlapping fields per optic nerve section were taken, resulting in a minimum of 6 images per mouse per antibody. A minimum of 5 animals per group were included in each study unless otherwise stated. Measurement of fluorescence intensity was performed using Image J software.

### 2.5. Analysis of Visual Function Recording

The function of retinal ganglion cells (RGCs) was examined using PERG with the Celeris Diagnosys pattern stimulator (Diagnosys, Lowell, MA, USA) available at the Augusta University Vision Core facility, following established protocols [51,52]. Pattern ERG (pERG) evokes electrical responses from the macular RGCs and has been utilized to diagnose several optic neuropathies and primary ganglion cell diseases [19,53]. The animals were dark-adapted overnight prior to the PERG experiment. Under dim red light, animals were anesthetized with intraperitoneal injections of a mixture of ketamine and xylazine. The corneas were treated with proparacaine (0.5%), and pupils dilated with topical phenylephrine HCl (2.5%) and tropicamide (1%). The mice’s head and neck were placed in a gently restraining custom-made holder that prevented position instability while the rest of the body lay on the heating table to maintain the body temperature at 37 °C. A combined light guide electrode and stimulator were placed on the cornea of one eye as the fellow eye reference electrode, and on the other eye was the pattern stimulator, both connected to the channel amplifier. A drop of Hypromellose was added to the cornea to improve electrical contact and protection from dryness. The PERG results from each eye were first recorded and then averaged across the two eyes of each mouse and across the mice in each group. Visual stimuli consisted of horizontal bars with a contrast of 99% at 0.155 cycles/degree spatial frequency 1 Hz temporal frequency. PERG waveforms consisted of three main waves: a negative wave (N1), a positive wave (P1) following the N1 with a peak latency of 90 to 100 ms, followed by a broad negative (N2) with peak latency in the range of 200 to 300 ms. The PERG data analysis was performed using custom Python scripts. The analysis pipeline encompassed data import and preprocessing to remove artifacts and noise, followed by signal averaging across both eyes to improve the signal-to-noise ratio. Statistical analyses, including descriptive statistics and comparative analyses, were performed using the SciPy scientific computing library. Matplotlib was utilized to generate figures illustrating the PERG waveforms and statistical results.

ERG recordings were performed in mice using the Touch/Touch feature of the Celeris Ophthalmic Electrophysiology System (Diagnosys, Lowell, MA, USA) available at the Vision Core, according to the published methods [54,55]. The Touch/Touch protocol stimulates one eye at a time and uses the fellow, unstimulated eye as the reference. For scotopic ERGs, mice were dark-adapted for approximately 16 h before the experiment, then tested using a series of light flashes of increasing energy (0.001, 0.005, 0.01, 0.1, 0.5, and 1.0 cd s/m^2^).

### 2.6. Statistical Analysis

Statistical analyses were performed using GraphPad Prism 9 (GraphPad Software Inc., La Jolla, CA, USA). One-way ANOVA followed by the Tukey test was used for multiple comparisons. A *p*-value < 0.05 was considered statistically significant.

## 3. Results

### 3.1. Spermine Oxidase Inhibition via MDL72527 Treatment Attenuates EAE-Induced Motor Deficits

**Figure 1:**
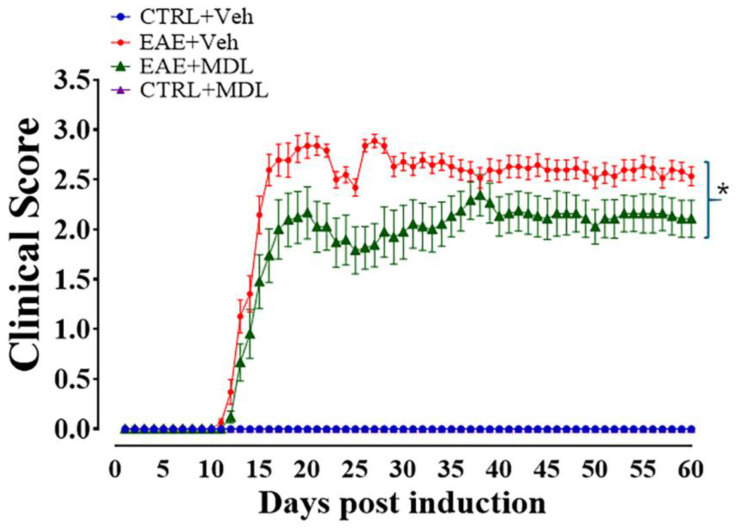
MDL72527 treatment attenuates EAE-induced motor deficits. Time course of clinical scores in EAE mice and their respective control groups. Vehicle-treated EAE mice (red line) show progressive worsening of motor function from Day 9 post-induction. However, MDL72527-treated EAE mice (green line) exhibit significantly lower clinical scores. The control groups displayed no motor deficits with scores parallel to the zero line. * *p* < 0.05 for days 21–27. *n* = 13–31 mice per group from three different experiments. Data presented as mean ± SEM. Clinical scores are based on a 0–5 scale as described in methods.

To evaluate the therapeutic effects of SMOX inhibition, we assessed their motor function over the subsequent 60 days (Figure 1), following methods previously published by our laboratory [42]. Animals were evaluated daily, and clinical symptoms were graded according to the scoring system described. The scoring scale demonstrates the progression of motor deficits, with higher scores indicating more severe impairment. The Veh-EAE mice group (red line, Figure 1) exhibited a progressive worsening of physical disability, marked by increasing clinical scores starting on Day 9 post-induction. The spike in the score graph reflects this during days 9–15, followed by a steady state of clinical symptom manifestation. In contrast, the MDL-EAE mice (green line, Figure 1) presented lower clinical scores, which were statistically significant (*p* < 0.05) during days 21–27. Likewise, the onset of motor symptoms in this mice group was delayed compared to the Veh-EAE group. The peak difference in clinical scores between the two groups was observed around Day 28, when the MDL-EAE group showed approximately a 40% reduction in clinical scores compared to the Veh-EAE group. Moreover, the severity of symptoms, as indicated by the clinical scores, was consistently lower in the MDL-treated mice for the duration of the experiment. It is noteworthy that the control mice groups (Veh-Con and MDL-Con), which did not undergo EAE induction, showed no signs of motor deficits and maintained a clinical score of 0 throughout the experimental period. In line with our previous study [42], this part of the current study provides additional evidence that inhibition of SMOX effectively attenuates the onset, progression, and severity of motor deficits in the EAE mouse model.

### 3.2. SMOX Inhibition Preserves Optic Nerve Myelin Thickness and Axonal Number in EAE Mice

**Figure 2:**
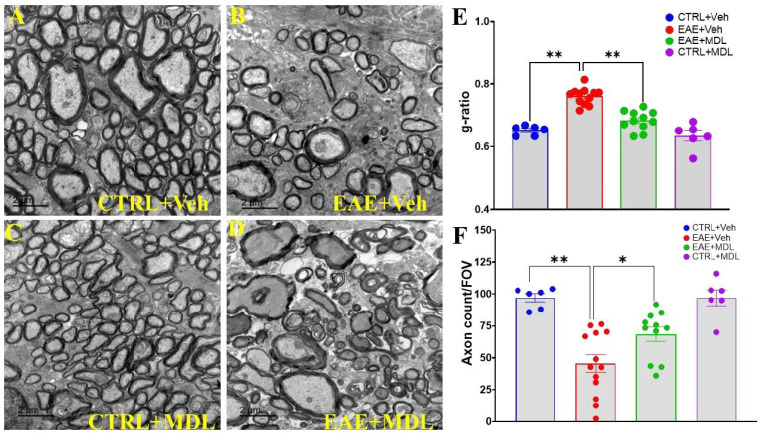
Spermine oxidase inhibition preserves optic nerve integrity in EAE mice. (**A**–**D**) Representative electron microscopy images of optic nerve sections from vehicle control, vehicle-treated EAE, and MDL72527-treated EAE mice. Vehicle-treated EAE mice show axonal loss and demyelination, while MDL72527 treatment markedly reduces these deficits. Scale bar: 2 µm. (**E**) G-ratios across groups. Vehicle-treated EAE mice show increased g-ratios compared to vehicle control, indicating demyelination. MDL72527 treatment significantly reduces this increase in EAE mice. (**F**) Axon count per field of view. Vehicle-treated EAE mice exhibit reduced axon counts, while MDL72527 treatment preserves axon integrity. Data presented as mean ± SEM. * *p* < 0.05, ** *p* < 0.01. *n* = 6–13 mice per group.

In this study, we investigated the impact of SMOX inhibition on demyelination in EAE. The electron micrographs of optic nerve cross-sections revealed clear and distinct differences among the groups. The Veh-control group (Figure 2A) exhibited the hallmarks of a healthy optic nerve: densely packed axons surrounded by thick, uniform myelin sheaths, consistent with normal neuroanatomy [56]. Conversely, the Veh-EAE mice (Figure 2B, top right) showed pathological changes, including a marked reduction in axon density and varying degrees of demyelination, including thinner or completely absent myelin sheaths, typical of the neurodegenerative processes seen in EAE and multiple sclerosis. However, in alignment with our hypothesis, the MDL-EAE mice (Figure 2D, bottom right) showed preservation of both axonal density and myelin integrity. Although some evidence of demyelination and axonal loss persisted, the overall appearance was markedly improved. Quantitative analysis of g-ratios and axon counts provided further evidence of these protective effects. The g-ratio analysis (Figure 2B), measuring the ratio of inner axonal diameter to the total outer diameter, revealed low g-ratios (0.65 ± 0.006) in Veh-Con groups, indicative of healthy, thick myelin sheaths. The Veh-EAE mice exhibited significantly increased g-ratios (0.76 ± 0.008), confirming substantial demyelination. MDL-treated EAE mice showed a marked reduction in g-ratios (0.683 ± 0.009) compared to Veh-EAE mice (*p* < 0.01), though still elevated compared to controls, indicating significant but incomplete preservation of myelin integrity. Furthermore, axon count analysis demonstrated a substantial reduction in Veh-EAE mice (45 ± 6.98/FOV), compared to the Veh-Con group (97 ± 3.25/FOV), and significantly higher counts in MDL-EAE mice (68 ± 5.69/FOV, *p* < 0.05), confirming the axonal protection in response to SMOX inhibition.

### 3.3. The Optic Nerve g-Ratios and Axon Diameter Relationship Is Altered in EAE

**Figure 3:**
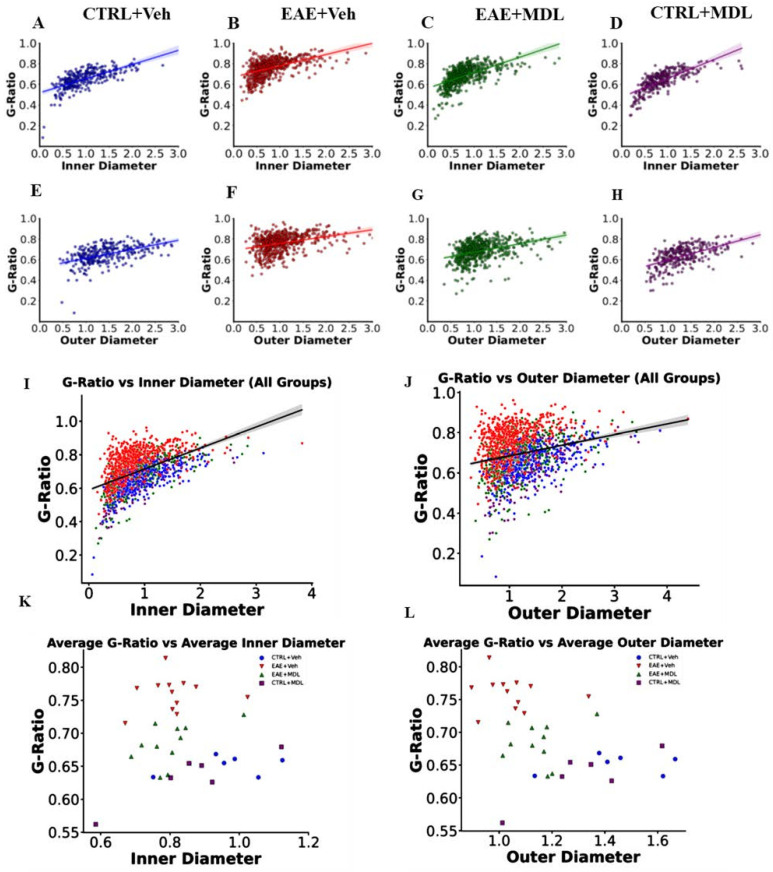
Axon diameter and g-ratio relationships across experimental groups: Regression plots of inner axon diameter (**A**–**D**) and outer axon diameter (**E**–**H**) against g-ratio for WT-CTRL (blue), WT-EAE (red), EAE-MDL (green), and CTRL-MDL (purple). Total g-ratios vs. axon diameter and average g-ratios per mouse across the four mice groups for inner diameter (**I**,**J**) and outer diameter (**K**,**L**) are shown. Linear regression analysis reveals alterations in both inner and outer axon diameter-g-ratio relationships in EAE and significant improvement with MDL72527 treatment. Inner diameter consistently shows stronger correlation with g-ratio across all groups. Data points represent individual axons; lines indicate linear regression fits. *n* = 6–13 mice per group.

Linear regression analysis was performed to examine the relationship between axon diameter and g-ratios across the different groups. Our results show significant alterations in the relationship between the two measures during EAE and following treatment with the SMOX inhibitor MDL72527. In Veh-Con mice (Figure 3A,G), we observed a positive correlation between axon diameter and g-ratio for both inner and outer diameters. The inner diameter showed a stronger correlation (slope = 0.137, R^2^ = 0.458, *p* < 0.01) compared to the outer diameter (slope = 0.088, R^2^ = 0.276, *p* < 0.01). This relationship indicates that in healthy conditions, larger axons tend to have proportionally thinner myelin sheaths, resulting in higher g-ratios. However, in Veh-EAE mice (Figure 3B,H), this relationship was significantly altered. While a positive correlation was still observed, the strength of this relationship decreased substantially, particularly for the outer diameter (inner: slope = 0.109, R^2^ = 0.266, *p* < 0.01; outer: slope = 0.066, R^2^ = 0.126, *p* < 0.01). The weakening of this correlation in EAE mice suggests a more complex relationship between axon diameter and g-ratio compared to healthy mice. Interestingly, treatment with MDL72527 in EAE mice (EAE-MDL, Figure 3C,I) considerably restored the relationship between axon diameter and g-ratio. The correlational strengths for both inner and outer diameters (inner: slope = 0.151, R^2^ = 0.362, *p* < 0.01; outer: slope = 0.084, R^2^ = 0.156, *p* < 0.01) were improved compared to the vehicle-treated EAE group, though not fully restored to control levels. The control group treated with MDL72527 (MDL-Con, Figure 3D,J) showed the strongest correlation between axon diameter and g-ratio (inner: slope = 0.182, R^2^ = 0.550, *p* < 0.01; outer: slope = 0.123, R^2^ = 0.343, *p* < 0.01). This enhanced correlation in healthy optic nerves treated with MDL72527 is a pointer to the beneficial effects that spermine oxidase inhibition may have on axon-myelin relationships, even in the absence of pathology.

### 3.4. SMOX Inhibition Preserves Myelin Proteins in the EAE Optic Nerve

**Figure 4:**
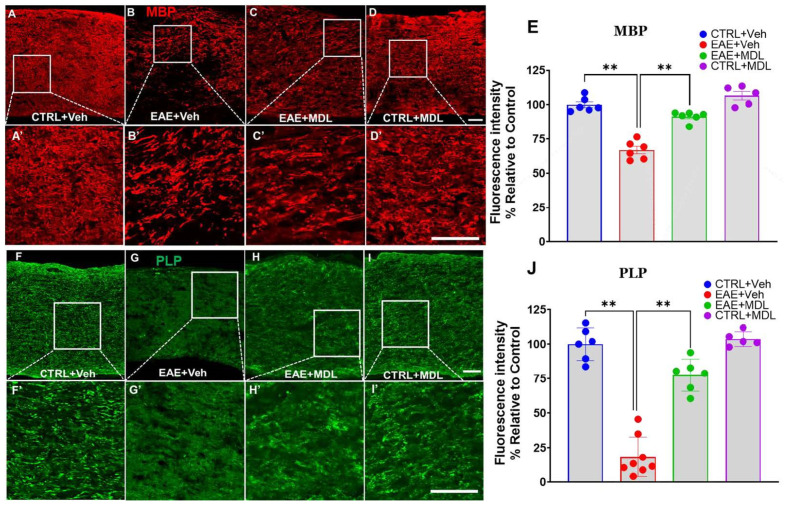
SMOX inhibition protects against myelin loss in EAE optic nerve. (**A**–**D**) Representative confocal images of optic nerve sections immunolabeled for myelin basic protein (MBP) in control, vehicle-treated EAE, and MDL72527-treated EAE mice. (**A’**–**D’**) Magnified images of the boxed regions demonstrating the changes. (**E**) Quantification of MBP immunofluorescence intensity across groups. (**F**–**I**) Representative confocal images of optic nerve sections immunolabeled for proteolipid protein (PLP) in control, vehicle-treated EAE, and MDL72527-treated EAE mice. (**F’**–**I’**) Magnified images of the boxed regions demonstrating the changes. (**E**) Quantification of MBP immunofluorescence intensity across groups (**J**) quantification of PLP immunofluorescence intensity across groups. Vehicle-treated EAE mice show substantial loss of both MBP and PLP, while MDL72527 treatment significantly mitigates this loss. Scale bar 50 µm. Data presented as mean ± SEM. ** *p* < 0.01. *n* = 5–8 mice per group.

Confocal images of optic nerve sections immunostained for myelin basic protein (MBP, Figure 4A–D) revealed significant differences between the groups. The Veh-EAE mice optic nerve samples showed a substantial reduction in MBP level in comparison to the uniform, intense MBP staining observed in healthy control (Veh-Con) mice. In contrast, optic nerves from EAE mice treated with the SMOX inhibitor MDL72527 showed a notable preservation of MBP. While some reduction in staining intensity was still evident compared to healthy controls, the overall MBP expression level in MDL72527-treated EAE mice was markedly stronger and more uniform than in the vehicle-treated EAE group. Quantitative analysis of MBP immunofluorescence intensity confirmed these visual observations (*p* < 0.01), indicating a significant protective effect of SMOX inhibition against EAE-induced myelin loss (Figure 4E). Furthermore, we also examined the expression of proteolipid protein (PLP), the most abundant myelin protein in nerve tissues (Figure 4F–I). Similar to the MBP expression, healthy control optic nerves showed strong, uniform PLP staining in the tissues. In contrast, optic nerves from vehicle-treated EAE mice revealed a marked reduction in PLP immunoreactivity, with notably less intense and severely discontinuous staining. EAE mice treated with MDL72527 demonstrated a reversal of this condition via significant preservation of PLP immunoreactivity compared to the vehicle-treated EAE group. Quantitative analysis of PLP immunofluorescence intensity yielded results consistent with the MBP findings (*p* < 0.01), further supporting the protective effect of SMOX inhibition against myelin loss in EAE optic nerves (Figure 4J).

### 3.5. EAE-Induced Axonal Damage Is Reduced by SMOX Inhibition

**Figure 5:**
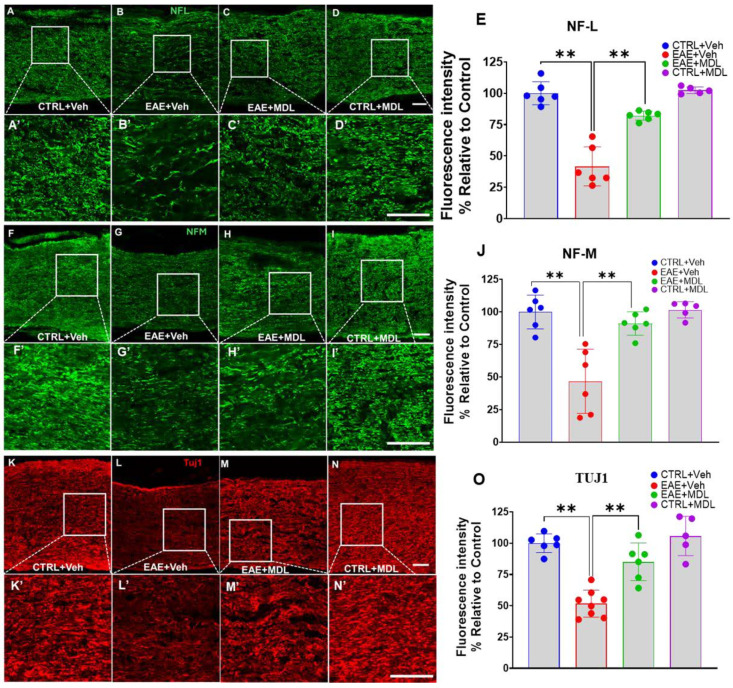
SMOX inhibition rescues EAE-induced axonal injury in the optic nerve. (**A**–**D**) Representative confocal images of optic nerve sections immune stained for Neurofilament-L (NF-L). (**A’**–**D’**) Magnified images of the boxed regions demonstrating the changes. (**E**) Quantification of NF-L immunofluorescence intensity. (**F**–**I**) Representative confocal images of optic nerve sections immune stained for Neurofilament-M (NF-M). (**F’**–**I’**) Magnified images of the boxed regions demonstrating the changes. (**J**) Quantification of NF-M immunofluorescence intensity. (**K**–**N**) Representative confocal images of optic nerve sections immune stained for beta-III tubulin (TUJ1). (**K’**–**N’**) Magnified images of the boxed regions demonstrating the changes. (**O**) Quantification of TUJ1 immunofluorescence intensity. EAE induces downregulation of axonal markers (NF-L, NF-M, TUJ1), which is significantly improved by MDL72527 treatment. Scale bar: 50 µm. Data presented as mean ± SEM. ** *p* < 0.01. *n* = 5–8 mice per group.

We continued our investigation of the neuroprotective effects of SMOX inhibition in EAE by further analyzing mediators of axonal integrity. Here, we examined three key neurofilament proteins—Neurofilament-L (NF-L), Neurofilament-M (NF-M), and beta-III tubulin (TUJ1)—using immunofluorescence staining of optic nerve sections. Figure 5 shows confocal images demonstrating considerable differences in the expression patterns of the axonal markers across the four mice groups. In the Veh-EAE mice, there is a marked reduction in NF-L staining, disparate and irregular, compared to the Veh-Con. On the other hand, the optic nerves from EAE mice treated with the SMOX inhibitor MDL72527 demonstrated notable preservation of NF-L expression. While some reduction in intensity was still evident compared to controls, the overall NF-L signal was substantially stronger and more uniform than is observed in the vehicle-treated EAE group. Quantitative analysis of NF-L fluorescence intensity (Figure 5E) confirmed these observations, with statistically significant improvement in NF-L expression levels in MDL72527-treated EAE mice compared to vehicle-treated EAE mice (*p* < 0.01). Similar patterns were observed for NF-M (Figure 5F–I) and TUJ1 (Figure 5K–N) immunostaining. In both cases, vehicle-treated EAE mice exhibited a substantially decreased NF-L expression compared to controls, a pointer to the ongoing widespread axonal damage associated with EAE disease progression. This pathological onslaught was reversed upon treatment with MDL72527, resulting in significant preservation of both NF-M and TUJ1 proteins in EAE mice. Quantitative analysis of the fluorescence intensities for NF-M (Figure 5J) and TUJ1 (Figure 5O) corroborated these visual findings (*p* < 0.01).

### 3.6. MDL72527 Treatment Rescues RGC Dysfunction in EAE Mice

**Figure 6:**
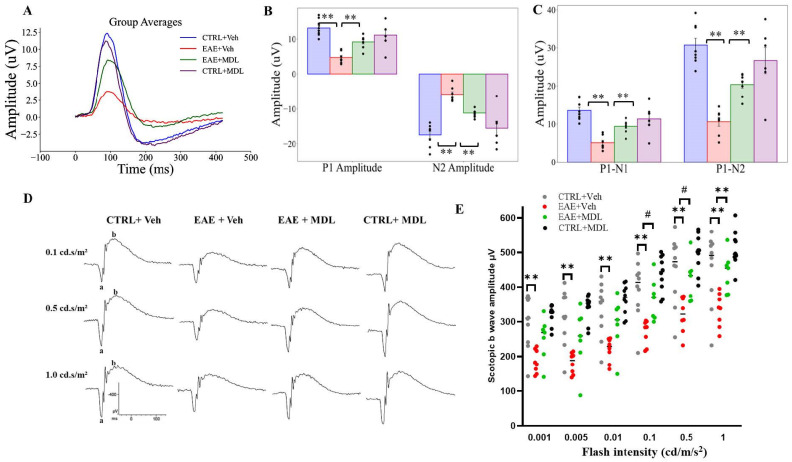
MDL treatment rescues PERG amplitude in EAE mice and restores RGC electrical signal strength in EAE mice. (**A**) Composite waveforms for all the mice groups are shown. PERG was recorded in response to reversing gratings (temporal frequency 1 Hz, spatial frequency 0.05 cycles/deg, contrast 1.0). (**B**) Bar chart displaying P1 and N2 amplitudes across different groups. (**C**) Bar graph illustrating P1-N1 and P1-N2 amplitudes showing significant differences between WT-EAE mice and MDL-treated groups. Data presented as mean ± SEM; ** *p* < 0.01. *n* = 7–8 mice per group. (**D**) Representative ERG tracings at various intensities. (**E**) Changes in scotopic b-wave amplitudes were studied at flash intensities ranging from 0.001 to 1.0 cd/s/m^2^. Data are shown as mean ± SEM. *n* = 7–8 per group. ** *p* < 0.01; ^#^ *p* < 0.05.

In this study, we employed pattern electroretinogram (PERG) to assess retinal ganglion cell function in EAE and the impact of SMOX inhibition on EAE-induced functional changes. Similar to previous reports [19,51,57], the PERG recordings were conducted on Day 17, the peak of EAE clinical symptoms. The PERG waveforms for the four groups (Figure 6A–D), with each group’s data displayed as individual waveforms for each eye and the grand average highlighted (Supplementary Figure S1). These waveforms represent the retinal response to reversing gratings with specific parameters (temporal frequency 1 Hz, spatial frequency 0.05 cycles/deg, contrast 1.0). Figure 6A shows the composite waveforms for each mice group, showing distinct patterns across the experimental conditions.

As presented in Figure 6B, the P1 mean amplitude, a key indicator of RGC function, showed marked variations between groups. WT-EAE mice exhibited significantly lower P1 amplitudes compared to both control groups and MDL-treated EAE mice (*p* < 0.01). This reduction in P1 amplitude in untreated EAE mice reflects severe impairment of RGC electrical responses, consistent with the neurodegenerative processes associated with EAE. Conversely, EAE mice treated with MDL-72527 demonstrated a significant rescue of the P1 amplitude (*p* < 0.01). SMOX inhibition effectively preserves RGC function in the face of EAE-induced damage. Control mice treated with MDL-72527 showed P1 amplitudes comparable to vehicle-treated controls, suggesting that the treatment does not adversely affect healthy RGCs. Moreover, analysis of the N2 mean amplitude (Figure 6B) supported these findings. WT-EAE mice showed a significant reduction in N2 amplitude compared to control groups, further confirming the detrimental effect of EAE on RGC function. MDL72527 treatment in EAE mice mitigated amplitude decrement, with N2 amplitudes significantly higher than those of untreated EAE mice (*p* < 0.01).

The P1-N1 mean amplitude, a crucial indicator of RGC signal strength, provides insight into the effects of SMOX inhibition on several aspects of visual functioning in mice. This parameter was evaluated, and differences of measure were noted across the experimental groups (Figure 6C). WT-EAE mice exhibited a substantial reduction (around 61%) in P1-N1 amplitude compared to the control group. In contrast, EAE mice treated with the SMOX inhibitor MDL-72527 demonstrated a significantly milder reduction, with P1-N1 mean amplitude decreased by only about 28% relative to controls. Similarly, in the analysis of the P1-N2 mean amplitude (Figure 6C), the WT-EAE mice group showed a 65% reduction in P1-N2 amplitude compared to the control group, again highlighting the severe impact of EAE on RGC function. However, in the MDL-72527 treated EAE mice group, P1-N2 amplitudes were reduced by 34% compared to controls. In both measures (P1-N1, P2-N2), MDL treatment resulted in the significant preservation of the signal strength lost in untreated EAE mice (*p* < 0.01). The PERG results obtained with the MDL72527 treated control are similar to the vehicle controls.

Results from the ERG experiments showed that scotopic b waves are impacted in EAE mice. Compared with Con-Veh mice, EAE Veh mice showed significantly reduced scotopic b- amplitudes at all intensities studied (0.001, 0.005, 0.01, 0.1, 0.5, and 1.0 cd s/m^2^), indicating the altered inner retinal function (Figure 6E,F). In response to SMOX inhibition using MDL72527 treatment in EAE mice, improvements in b-amplitudes were observed at all intensities with significant increases at intensities 0.1, 1.0, and 5.0 cd s/m^2^). No changes were observed in a-waves between the two EAE groups. The responses between the Con Veh and Con MDL groups were comparable. At the same time, changes in a-wave were not markedly changed.

### 3.7. SMOX Inhibition Suppresses EAE-Induced Upregulation of Galectin 3

**Figure 7:**
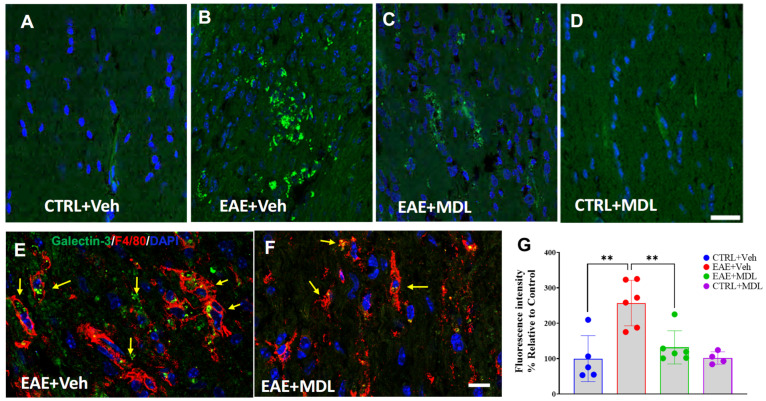
SMOX inhibition exerts anti-inflammatory effects in EAE disease. (**A**–**D**) Representative confocal images of optic nerve sections stained with galectin-3 antibody in control, vehicle-treated EAE, and MDL72527-treated EAE mice. (**E**,**F**) Gal3 and F4/80 staining on optic nerve sections from Veh EAE and MDL EAE mice showed co-localization, as indicated by the arrows. Scale bar 50 µm. (**G**) Quantification of galectin immunofluorescence intensity across the groups. Data presented as mean ± SEM. ** *p* < 0.01. *n* = 4–6 mice per group.

We further investigated the underlying mechanisms of neuroprotection offered by SMOX inhibition. Figure 7 illustrates the distinct Galectin 3 (Gal3) expression patterns among control, vehicle-treated EAE, and MDL72527-treated EAE mice groups (Figure 7A–D). We observed a minimal expression of Gal3 in the Veh-Con and MDL-Con optic nerves. In contrast, Veh-EAE optic nerve sections exhibited markedly increased Gal3 immunoreactivity (indicated by arrows). Interestingly, SMOX inhibition attenuated this increase, as evidenced by reduced Gal3 levels in MDL-EAE samples. Quantitative analysis of Gal3 immunofluorescence intensity corroborated these observations, demonstrated by the significant differences (Figure 7G). The Veh-EAE group showed significantly elevated Gal3 expression compared to the control group (*p* < 0.01). Conversely, MDL72527-treated EAE mice exhibited significantly reduced Gal3 immunofluorescence intensity relative to the Veh-EAE group (*p* < 0.01). To further pinpoint the cellular source of Gal3, we performed a Gal3 and F4/80 double staining for microglia/macrophages. The co-localization of Gal3 and F4/80 was evident in the EAE optic nerve sections (Figure 7E,F), confirming that the major Gal3 expression corresponds to macrophage/microglia in EAE optic nerves.

## 4. Discussion

The present study explored the therapeutic potential of targeting SMOX with the pharmacological inhibitor MDL72527, providing strong evidence for neuroprotective benefits in a chronic mouse model of MS. SMOX inhibition was shown to attenuate EAE-induced motor deficits, reduce optic nerve myelin and axonal damage, and preserve RGC function. While our previous research examined how SMOX inhibition ameliorates several EAE pathologies, our current study offers a novel and comprehensive examination of the effects of SMOX inhibition on the visual system in EAE. Our multi-faceted approach assessed the impact of SMOX inhibition on disease progression through clinical scoring of motor deficits, ultrastructural analysis of myelin integrity and axonal preservation, immunohistochemical assessment of inflammatory markers, myelin and axonal proteins, and functional evaluation of RGCs, providing direct evidence and valuable new insights into the neuroprotective role of SMOX inhibition. These results highlight the potential of SMOX inhibition as a therapeutic strategy for vision loss in MS patients.

The marked reduction in clinical scores and delayed onset of motor impairment in MDL72527-treated EAE mice are consistent with the findings from our previous studies [42]. Although physical disability is a primary indication of disease progression in EAE [20], motor impairment in MS is a consequence of nervous deterioration diagnosed as the presence of white matter plaques in the brain [2,9,58,59]. Similarly, chronic EAE is underlined by a persistent loss of myelin and axonal proteins [9,59]. Results from the current study link the ameliorative effects of SMOX inhibition on EAE-induced physical disability to the preservation of myelin thickness and axonal integrity. Consistent with previous studies [60,61,62,63], optic nerve demyelination was evident in EAE mice in our study. However, in support of our hypothesis, electron microscopy results revealed significant preservation of the axon-myelin unit in MDL72527-treated mice. Corroboratively, the increased expression of PLP and MBP, the most abundant myelin proteins [64,65], as well as axonal neurofilaments (NF-L & M and Tuj1) were also evident in response to SMOX inhibition in our model. These results further demonstrate the therapeutic effectiveness of SMOX inhibition for preserving vision in MS pathology.

Following the established outcomes of several studies, where g-ratio was utilized as an objective measure of myelin loss and recovery [66,67], we analyzed the same using optic nerve electron microscopy. As expected, optic nerves from Veh-EAE mice were significantly demyelinated, indicated by higher g-ratios. This contrasts with the considerably lower g-ratios and higher axon counts in MDL72527-treated EAE mice. Given that g-ratios reflect a stable relationship between the inner and outer diameters of axons [68,69], which is destabilized in EAE and MS patients [68,69,70], we performed regression analysis to explore the relationships between the g-ratios and both inner and outer diameters in EAE. We hypothesized that under EAE pathological conditions, where demyelination and axonal damage are prevalent [59,71], the normal relationship between g-ratio and axon/fiber diameters observed in healthy tissues will be distorted, resulting from the demyelination and axonal degeneration [72] derailing the structural regularity of the nerve fibers. Our study concludes that SMOX inhibition with MDL72527 in EAE mice preserves myelin and axonal structure, as evidenced by lower g-ratios and higher axon counts, contrasting with the disrupted g-ratio and axon/fiber diameter relationship seen in demyelinated, vehicle-treated EAE mice.

In this study, we uncovered intriguing dynamics between axon size and myelin thickness across different experimental conditions. In the control mice, a positive correlation was observed between axon diameter and g-ratio, with larger axons tending to have proportionally thinner myelin sheaths. In particular, alterations of the inner diameter are more strongly correlated with g-ratios compared to the outer diameter. Early investigations of MS lesions show that the reduction in axonal density and transected axons are perennial features of chronic MS [73,74]. This is equally the case in late EAE, where significant loss of postsynaptic and myelin proteins have been observed in the retina and optic nerve [75]. Hence, the prevailing consensus is that neurodegeneration is the major cause of irreversible neurological damage in MS [76]. This is supported by the results of the quantitative analysis of g-ratio measurements we carried out on the EAE optic nerves. Changes in the inner axonal diameter have a greater impact on the g-ratio. This relationship was significantly disrupted in EAE mice, showing a weak correlation between g-ratio and axon diameters, indicating abnormal axon-myelin ratios. As expected, SMOX inhibition with MDL72527 restored a stronger, more positive association between g-ratios and axon diameters. This finding supports the role of SMOX in not only neuroprotecting myelin thickness but also maintaining the physiological scaling of myelin to axon size. We recognize that previous studies have demonstrated a strong correlation between axon size and myelin thickness, particularly the relationship between axon caliber, g-ratio, and broader metrics like myelin volume fraction [70,77,78]. However, this study is the first to examine the relationship between g-ratio and inner/outer axon diameter under conditions of optic nerve demyelination and axonal damage in an EAE model. We consider our findings to be significant, especially in EAE, where the correlation between outer diameter and g-ratio diminishes compared to control groups. The maintenance of a stronger correlation with inner diameter than the outer diameter in EAE emphasizes the effect of axonal degeneration, condensation, density loss, and swelling on g-ratio, even in the presence of other visual pathologies [72,73,76,79]. These present a strong case for the critical importance of axonal integrity in maintaining a stable g-ratio, especially in neuroinflammatory conditions such as MS/optic neuritis.

The structural perturbations observed in the optic nerve axons were further investigated using electrophysiological studies, which evaluated visual function across the different groups of mice. The non-invasive PERG technique, which is increasingly utilized in mouse models of optic nerve diseases like glaucoma and optic neuritis, offers a sensitive method for monitoring RGC function over time, thus enhancing our understanding of neurodegenerative processes and the effects of neuroprotective treatments in MS and related conditions [51,52]. In humans, PERG studies show that reduced P50 and N95 amplitudes reflect structural abnormalities in the macular region and RGCs [80]. Similarly, selective ganglion cell lesions in mice eliminate PERG recordings but do not flash ERG, thereby emphasizing PERG’s specificity in capturing RGC function [19]. Surprisingly, despite the importance of PERG-visual functioning tests, only a few studies [81,82] have employed PERG to evaluate the link between RGC function and motor disability in EAE mice. This gap may be due in part to the limited number of studies investigating visual function in mice post-EAE induction. However, such tests are essential for evaluating the extent of visual dysfunction consequent to optic nerve damage, as demonstrated in this study. As we have shown, the significant improvement in pattern electroretinogram (PERG) responses was evident in MDL72527-treated EAE mice, indicating that SMOX inhibition effectively preserves RGC function. Moreover, the rescue of P1 and N2 amplitudes, as well as P1-N1 and P1-N2 mean amplitudes, outlines the protective benefits of SMOX blockade against EAE-induced visual dysfunction at multiple levels of retinal processing such as RGC excitation and recovery. The functional data support our previous findings, complement the current results, and confirm our understanding of the therapeutic potential of SMOX inhibition [81]. These findings corroborate other studies showing the importance of preserving RGC function in MS and its animal models. Further, the results from the ERG analysis support the preservation of inner retinal function in EAE in response to SMOX inhibition. Reduced a- and b-wave amplitudes have been reported in EAE mice, suggesting the involvement of other retinal neurons and Müller glia in visual dysfunction. Additionally, thinning of the inner plexiform layer and synaptic loss have been observed in both EAE mice and MS patients [83,84,85]. The difference in a-amplitude observed in our study compared to others could result from the changes in the time of the disease and flash intensities applied. The observed gain in the positive deflection of the ERG b-wave in response to SMOX inhibition provides compelling evidence for the protective effects of SMOX inhibition on retinal physiology. These findings demonstrate that MDL-treated EAE mice possess improved functioning of rod bipolar and Müller cells, which play a crucial role in maintaining retinal health and signal transmission [86,87,88]. Interestingly, this shapes our interpretation of earlier findings on RGC function in MDL-treated EAE mice, suggesting that the improvement of RGC functioning stems not only from enhanced axonal integrity but also from preserved rod cell communication with the inner plexiform layer, which is crucial for efficient visual signal processing. While these findings suggest that SMOX inhibition exerts a broad protective influence on retinal physiology, further studies are needed to characterize the impact of SMOX inhibition on bipolar cells, photoreceptors, and Müller glia in the EAE retina to determine their contribution to overall retinal function.

Since neuroinflammation is a staple of EAE/MS disease [89,90], we further investigated the effects of SMOX inhibition on mediators of inflammation. Our previous study have demonstrated a significant reduction in optic nerve inflammation in response to SMOX inhibition [42]. In the present study, we sought to identify the mediators of SMOX-induced neuroinflammation. Our investigation reveals a novel connection between SMOX inhibition and Gal3 expression, establishing that SMOX inhibition leads to the downregulation of Gal3 in EAE optic nerves. This suggests that Gal3 may act as a downstream effector in SMOX-mediated neuroinflammation. Recent research has highlighted Gal3 as a crucial regulator of inflammation in various neurodegenerative conditions, including MS and EAE [91,92,93]. By interacting with TLR4 and TREM2, Gal3 is a key modulator of microglia activation in neurodegenerative states [94,95]. However, it also adds a degree of complexity to our understanding of Gal3’s involvement, as some studies have uncovered the anti-inflammatory effects of Gal3 [96,97]. We propose that further research will be required to fully identify the exact roles of Gal3 in MS and EAE and its regulation via SMOX. The diminished population of Gal3 and F4/80 double-positive cells in MDL72527-treated EAE mice, compared to the Veh-EAE group, further pinpoints microglia/macrophages as key cellular mediators of SMOX-related neuroinflammation. This is consistent with our earlier findings on the suppression of Iba1 and F4/80 positive cells in response to SMOX inhibition [42] as well as with SMOX’s role as a regulator of inflammation in other pathologies [98,99,100]. In our previous investigation, we observed changes in acrolein conjugates, a mediator of oxidative damage, in response to SMOX inhibition. It is worthwhile to explore whether acrolein contributes to the upregulation of Gal3 in EAE. These studies are under investigation. Studies have indicated the role of SMOX signaling in neuroprotection through various pathways, including antioxidant signaling via Nrf2 in experimental stroke [101,102], neuroinflammation in cerebral ischemia via IL6/TNFa [39], and miR-340-5p/Smurf1 signaling in spinal cord injury [103]. Supplementation of spermidine, a product of SMOX activity, demonstrated neuroprotective effects in Alzheimer’s disease [104], aging [105,106], neuropathy [107], optic nerve injury [108], etc.

While the present study has provided novel and valuable insights into the mechanisms of optic neuritis in MS, there are limitations to this study. SMOX inhibition did not confer full protection against EAE pathology, even though there is a significant difference in clinical outcomes and the results of the histological investigations. This may be due to MDL72527’s inhibitory effect on multiple steps in the polyamine pathway, potentially leading to unintended cellular damage and exacerbating inflammation. This is common to therapeutic agents with multiple points of pharmacological action [109,110]. In the present study, we employed MDL72527 treatment as an approach to explore the therapeutic potential of SMOX signaling in the context of EAE/MS. MDL72527 has been utilized in a variety of pathological conditions, including cancer and inflammatory diseases [111,112,113]. Furthermore, other studies similar to ours have utilized this compound as a pharmacological irreversible inhibitor of SMOX to evaluate the impact of SMOX blockade on CNS disease progression [44]. For intervening SMOX activity, the present study did not incorporate other approaches beyond pharmacological inhibition. Importantly, this study primarily examined the neuroprotective effects of MDL72527 in a preventative setting without assessing its potential to reduce neurodegeneration and inflammation after the onset of optic neuritis. Further research is required to evaluate the therapeutic benefits of SMOX inhibition in treating established disease pathologies. The specific biomolecular pathways by which the inhibition of SMOX activity reduces neuroinflammation and neuronal damage are yet to be fully understood. Our ongoing and future investigations involve the genetic modulation of SMOX (through both overexpression and knockout) for detailed transcriptomic and proteomic analyses. A plethora of preclinical studies aiming for neuroprotective and neurodegenerative therapeutic developments are progressing [114]. These approaches include targeting myelin formation via lipid metabolism [115,116], mesenchymal and stem cell transplantation [117,118], and anti-LINGO therapies for remyelination [119]. Nevertheless, targeting SMOX offers a new and unique approach via anti-inflammatory and neuroprotective effects, which, based on the evidence we have provided in our studies, complements these studies.

## 5. Conclusions

In summary, this study provides compelling evidence for the neuroprotective effects of SMOX inhibition in EAE-induced optic neuritis. Through a comprehensive experimental approach, we demonstrated that SMOX inhibition enhances the preservation of myelin and axonal viability, maintains RGC function, and modulates neuroinflammatory responses. Our findings suggest that SMOX inhibitors hold significant promise as a disease-modifying therapy, capable of preventing the onset of optic neuritis and mitigating further neuronal damage in cases of mild disease. These results not only emphasize the potential of SMOX inhibition as a therapeutic strategy for vision loss associated with MS but also open new avenues for understanding the mechanisms of neuroinflammation and neurodegeneration in EAE/MS. Our findings may pave the way for future research into polyamine metabolism in neuroinflammatory diseases and the development of therapies targeting SMOX in human clinical trials. Furthermore, this research highlights the importance of targeting SMOX as a strategy for neuroinflammation, with potential implications for other neurodegenerative conditions marked by oxidative stress and inflammation.

## Figures and Tables

**Table 1:** List of antibodies used in the study.

Primary Antibody	Species	Dilution	Source/Catalog No
Myelin basic protein (MBP)	Rat	1:200	EMD-Millipore/MAB386 (Burlington, MA, USA)
Proteolipid protein (PLP)	Chicken	1:200	Novus Biological/Nb100-1608 (Centennial, CO, USA)
Neurofilament L (NF-L)	Rabbit	1:100	EMD-Millipore/AB9568 (Burlington, MA, USA)
Neurofilament M (NF-M)	Chicken	1:100	EMD-Millipore/AB5735 (Burlington, MA, USA)
anti-βIII-tubulin (TUJ1)	Mouse	1:200	Biolegend/801202 (San diego, CA, USA)
F4/80	Rat	1:200	Abcam/ab6640 (Waltham, MA, USA)
Galectin3 (Gal3)	Rabbit	1:150	Lifespan Biosciences/C357474 (Newark, CA, USA)
**Secondary Antibody**		**Dilution**	**Catalog No**
Donkey anti-rabbit		1:500	Invitrogen (A21206) (Carlsbad, CA, USA)
Donkey anti-rat		1:500	Invitrogen (A21208) (Carlsbad, CA, USA)
Donkey anti-mouse		1:500	Invitrogen (A31570) (Carlsbad, CA, USA)
Donkey anti-chicken		1:500	Invitrogen (A11039) (Carlsbad, CA, USA)

## Data Availability

All the data have been included in the manuscript.

## Supplementary Materials

The following supporting information can be downloaded at: https://www.mdpi.com/article/10.3390/biom15020158/s1, Figure S1: Pattern Electroretinogram Waveforms in control and experimental groups.
