# Supplementary material for: Targeting SMOX Preserves Optic Nerve Myelin, Axonal Integrity, and Visual Function in Multiple Sclerosis
Source: Biomolecules. 2025 Jan 21;15(2):158. doi: 10.3390/biom15020158 (PMC11853291; doi:10.3390/biom15020158)
Supplement: Supplementary file 1 [file biomolecules-15-00158-s001.zip › biomolecules-3390786-supplementary.pdf]

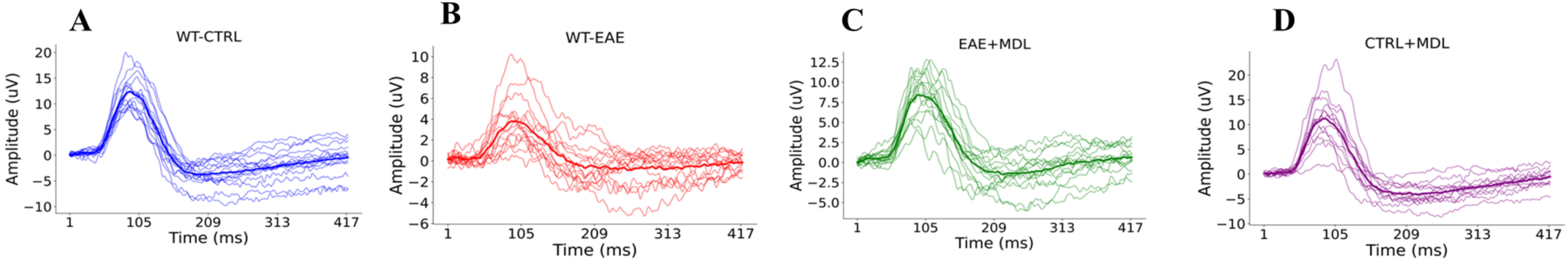

Figure S1: Pattern Electroretinogram Waveforms in Different Experimental Groups

(A-D) PERG waveforms recorded on Day 17 of the EAE disease model for each group: (A) WT-CTRL (B) WT-EAE (C) EAE+MDL (D) CTRL+MDL Each thin line represents an individual eye's response; bold lines show the grand average for each group. PERG recorded in response to reversing gratings (temporal frequency 1 Hz, spatial frequency 0.05 cycles/deg, contrast 1.0). N=7-8 mice per group.
